# Thickness-driven modulation of linear and nonlinear optical properties in La–Al co-doped ZnO thin films for optical limiting applications

**DOI:** 10.1039/d6ra00168h

**Published:** 2026-05-08

**Authors:** Preethika, Raghavendra Bairy

**Affiliations:** a Nitte (Deemed to be University), NMAM Institute of Technology (NMAMIT) Nitte, Department of Physics, Nanoscience Research Laboratory Karkala 574110 Karnataka India rbairy@nitte.edu.in rbairy@gmail.com

## Abstract

The influence of film thickness on the structural, surface, optical, and nonlinear optical properties of Al–La co-doped ZnO thin films was systematically examined. By adjusting the number of deposition cycles, film thicknesses in the ranges of 200–300 nm, 400–500 nm, and 700–800 nm were obtained. Powder X-ray diffraction (PXRD) analysis confirmed a hexagonal wurtzite crystal structure. Slight shifts in the (002) peak position with increasing film thickness indicated lattice parameter changes, and the decrease in peak width suggested improved crystallinity. Field emission scanning electron microscopy (FESEM) micrographs revealed that larger grain sizes developed as the films became thicker. Correspondingly, the surface roughness increased from 2.61 nm to 3.91 nm. The optical band gap exhibited a shift from 3.21 eV to 3.34 eV with increasing thickness. Z scan technique demonstrated that La–ZnO films possess self-defocusing characteristics, a positive nonlinear refractive index, and reverse saturable absorption (RSA). The third-order nonlinear optical susceptibility of the film increased from 200–300 nm to 400–500 nm thickness and decreased at 700–800 nm thickness due to the increased scattering and saturation effects associated with larger grain sizes and surface roughness at greater thicknesses, which can limit the efficiency of nonlinear interactions. Among the studied thicknesses, the 486 nm film exhibited the highest third-order nonlinear optical susceptibility, indicating that this thickness offers an optimal balance of crystallinity, grain size, and surface morphology for enhanced nonlinear optical performance.

## Abstract 2

The influence of film thickness on the structural, surface, optical, and nonlinear optical properties of Al–La co-doped ZnO thin films was systematically examined.

## Graphical abstract

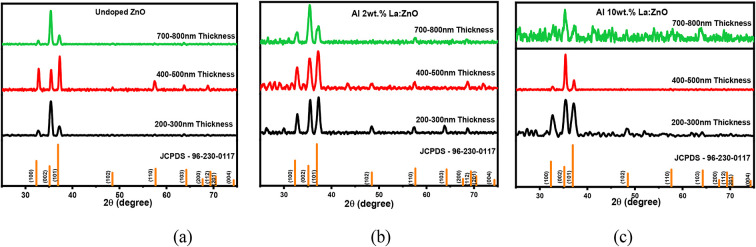

## 1. Introduction

Transparent conducting oxides (TCOs) have attracted significant research interest due to their distinctive combination of optical transparency and nonlinear optical properties, making them suitable for a wide range of commercial applications. This class of materials includes various metallic oxides such as indium oxide, tin oxide, indium tin oxide (ITO), zinc oxide (ZnO), cadmium–indium oxide, and cadmium–tin oxide. Among these metallic oxides, ZnO stands out as a versatile material with a hexagonal wurtzite crystal structure. It exhibits semiconducting, piezoelectric, and optical waveguiding properties, making it suitable for use in gas sensors,^1^ surface acoustic wave devices,^2^ transparent conductive electrodes,^3^ and photovoltaic applications.^4,5^ Zinc oxide (ZnO) has garnered considerable attention as a potential material for blue and ultraviolet light-emitting devices, primarily due to its wide band gap of approximately 3.3 eV and a high exciton binding energy of 60 meV. Lanthanum (La) doping can alter optical (linear and nonlinear) and electrical properties of ZnO. By shifting electronic states within the band structure with incorporating La^3+^ ions, can improve optical transparency, and enhances luminescence. Various fabrication techniques have been explored for ZnO thin films, including sputtering,^4^ metal–organic chemical vapor deposition (MOCVD),^5^ sol–gel processing,^6^ and spray pyrolysis.^7^ Among these methods, spray pyrolysis stands out for its simplicity, cost-effectiveness, and suitability for coating large surface areas. Typically, this technique involves spraying solutions such as zinc acetate dihydrate (Zn(CH_3_COO)_2_·2H_2_O), lanthanum chloride heptahydrate (LaCl_3_·7H_2_O) and aluminium chloride hexahydrate (AlCl_3_·6H_2_O) at a 0.1 molar concentration dissolved in a solvent. This study investigates how varying the thickness of ZnO thin films influences their surface morphology, as well as their structural and linear and nonlinear optical characteristics.

Previous studies have shown that the thickness of ZnO thin films significantly influences their crystalline quality as well as their electrical and optical properties. Myoung *et al.*^8^ highlighted the dependence of these properties on film thickness, while Reddy *et al.*^9^ investigated how structural, surface morphological, and optical characteristics vary in ZnO films fabricated *via* RF magnetron sputtering. In optoelectronic device applications, selecting an optimal film thickness is crucial for achieving maximum performance. Hence, understanding how thickness impacts film properties is essential. In this context, the present work focuses on examining the influence of film thickness on the structural, morphological, and optical behaviour of Al and La doped ZnO thin films deposited on glass substrates using the spray pyrolysis method.

## 2. Experimental details

ZnO thin films were deposited using the spray pyrolysis technique at a substrate temperature of 723 K. A 0.1 M solution of zinc acetate [Zn(CH_3_COO)_2_], prepared in deionized water, was used as the precursor. During the deposition, the nozzle was positioned 20 cm away from the substrate, and the solution flow rate was maintained at 500 µL min^−1^. Air served as the carrier gas at a constant pressure of 6 kg cm^−2^. As the aerosol droplets approached the heated substrate, pyrolytic decomposition occurred, resulting in the formation of well-adhered ZnO films.

ZnO films of various thicknesses were synthesized by keeping all spray parameters constant and varying only the deposition time. The thickness and surface roughness of the films were measured using a stylus profilometer. Structural properties were analyzed by X-ray diffraction (XRD) using a Rigaku diffractometer with Cu Kα radiation (*λ* = 1.5406 Å). Crystallite sizes were estimated using the Scherrer formula based on the broadening of XRD peaks. Optical characterization of the ZnO films was conducted at room temperature using a Shimadzu UV-1700 spectrophotometer across a wavelength range of 300–1100 nm. Additionally, the elemental composition of the ZnO films was determined through energy-dispersive X-ray analysis (EDX) coupled with a field emission scanning electron microscope (Carl Zeiss model). In this study, the photoluminescence (PL) analysis was performed using a Bruker Alpha II model. To evaluate the nonlinear optical properties of the material, the Z-scan technique was employed. This single-beam method, originally introduced by Sheikh Bahae,^22^ allows for the determination of both the nonlinear absorption (NLA) amplitude and the sign and strength of nonlinear refraction (NLR). The technique is particularly useful for estimating the material's nonlinear optical susceptibility, encompassing both its real and imaginary components.

## 3. Results and discussion

### 3.1 Structural properties- PXRD analysis

**Fig. 1:**
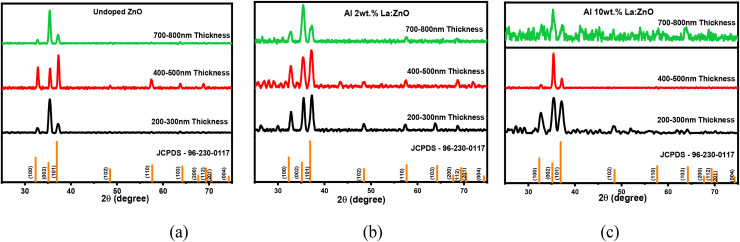
X-ray diffraction patterns of (a) undoped, (b) Al 2 wt% La:ZnO and (c) Al 10 wt% La:ZnO thin films.

The structural characteristics and preferred orientation of the ZnO thin film were examined using X-ray diffraction (XRD) analysis. The XRD patterns presented in Fig. 1 confirm the polycrystalline nature of the film. X-ray diffraction (XRD) analysis was performed to investigate the influence of film thickness on the structural properties of aluminium (Al) and lanthanum (La) co-doped ZnO thin films. Three distinct film thickness ranges, namely 200–300 nm, 400–500 nm, and 700–800 nm, were examined, with La concentration varying from 2 wt% to 10 wt% while Al content remained constant at 4 wt%. The XRD patterns of 4, 6, and 8 wt% Al–La co-doped ZnO thin films with varying thicknesses are presented in Fig. S1.

All samples consistently exhibited diffraction peaks characteristic of the hexagonal wurtzite ZnO structure (JCPDS-96-230-0117), with a prominent (002) peak indicating a preferential *c*-axis orientation perpendicular to the substrate. Additionally, the patterns reveal the presence of other orientations such as (101), along with lower intensity peaks corresponding to the (100), (103), (102), and (110) planes. No secondary phases corresponding to Al-oxides or La-oxides were detected, suggesting the successful incorporation of both dopants into the ZnO lattice.

The crystallite grain size was determined using the Scherrer formula,^10^1
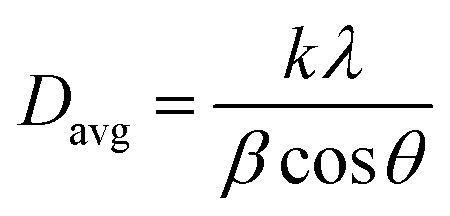
where *D* represents the crystallite size, *λ* (1.5405 Å) is the wavelength of the X-rays used, *β* is the full width at half maximum (FWHM) of the diffraction peak measured in radians, and *θ* is the Bragg angle.

A detailed comparison of the XRD patterns across the different thicknesses revealed a significant dependence of crystalline quality on film thickness. The 200–300 nm thickness films generally presented broader and less intense peaks, indicative of smaller crystallite sizes and a higher degree of structural disorder. Films with a thickness of 400–500 nm consistently exhibited the sharpest and most intense (002) diffraction peaks, signifying superior crystallinity, larger average crystallite sizes, and a more well-ordered structure. This suggests that 486 nm represents an optimal thickness where the film has grown sufficiently to overcome initial imperfections. Further increasing the film thickness to the 700–800 nm range did not yield additional improvements in crystalline quality; instead, the peaks for these thicker films were often comparable to or slightly less defined than those of the 400–500 nm range thickness films, particularly at higher La concentrations. This observation implies that, beyond an optimal thickness, factors such as accumulated intrinsic stress or growth-related defects might begin to impede further enhancement of crystallinity.^11^ Therefore, for the Al (4 wt%) and La co-doped ZnO system, a film thickness of approximately 400–500 nm appears to be critical for achieving the optimal structural characteristics. The crystallite size was calculated using the Scherrer equation. The error in the crystallite size is mainly due to determination of the full width at half maximum (FWHM) and instrumental broadening. Considering these factors, the estimated error in the calculated crystallite size is approximately ±2–3 nm. The crystallite sizes of Al:LZO thin films of different thicknesses are tabulated in Table 1.

**Table 1:** Crystallite size analysis for Al:LZO thin films

Sample composition wt%	2*θ* (deg) (002)	Avg. crystallite size *D* (nm)		
		200–300 nm	400–500 nm	700–800 nm
Undoped ZnO	35.377	21.78	24.39	18.01
Al 2 wt% LZO	35.403	19.01	23.43	18.66
Al 4 wt% LZO	35.396	17.64	23.16	18.67
Al 6 wt% LZO	35.399	23.38	28.18	20.09
Al 8 wt% LZO	35.392	11.95	20.01	20.81
Al 10 wt% LZO	35.522	11.47	22.61	21.22

A gradual shift of the ZnO (002) diffraction peak toward higher 2*θ* values was detected as the film thickness increased, suggesting the presence of compressive strain within the films. This strain was found to diminish as the thickness increased from 200–300 nm to 700–800 nm, which can be attributed to the enhancement in crystalline quality.^12^

The full width at half maximum (FWHM) values observed in the materials show a gradual rise with the incorporation of aluminium and lanthanum in higher thicknesses, indicating a reduction in the crystallinity of the thin films. This trend can be attributed to lattice strain induced by the ionic radius mismatch between the Zn and Al atoms; this may also be associated with the potential segregation of dopant atoms along the grain boundaries at higher doping concentrations. The microstructural parameters, namely dislocation density (*δ*) and lattice strain (*ε*), were evaluated using the following relations.^13^2
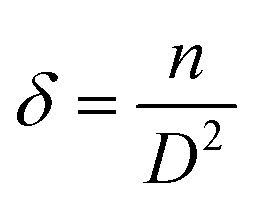
3
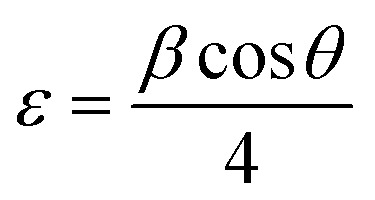
Here, *n* is constant (*n* = 1), *D* is the average crystallite size, *β* represents the FWHM and *θ* is the Bragg angle. A rise in dislocation density generally signifies an increase in defects and distortions within the crystal lattice. The formation of dislocations, particularly at grain boundaries, contributes to the development of internal strain. Experimental findings given in Table 3 reveal that as the dopant concentration in the films rises, both dislocation density and internal stress also increase, indicating a higher defect density This behaviour is attributed to the substitution of Zn atoms by dopant La and Al atoms, which introduces lattice distortion and, consequently, enhances micro strain in the deposited thin films.

The lattice parameters were determined using the following formula.^14^4
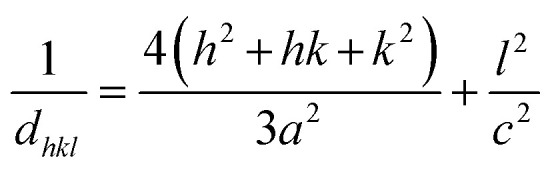


The lattice parameters *a* and *c* were calculated using the following expressions.^15^5
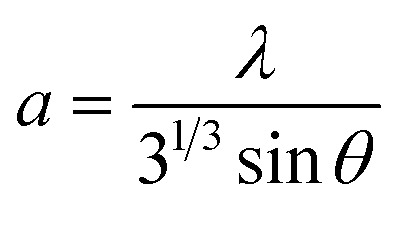
6
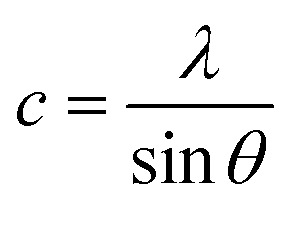
The observed changes in the lattice constants *a* and *c* suggest that the increasing thickness of the films leads to slight structural contraction. The obtained values were *a* = 3.21907 Å and *c* = 5.15760 Å, which are in close agreement with the standard values reported in the JCPDS database, confirming the high quality and hexagonal structure of the ZnO film (Table 2).

**Table 2:** PXRD results for Al:LZO thin films

Material composition Al:LZO (wt%)	2*θ* (deg.) (002)			FWHM (β)			*d*-Spacing (Å) *d*_(*hkl*)_		
	200–300 nm	400–500 nm	700–800 nm	200–300 nm	400–500 nm	700–800 nm	200–300 nm	400–500 nm	700–800 nm
0 wt%	35.39	35.37	35.73	0.382	0.341	0.415	2.530	2.535	2.536
2 wt%	35.67	35.40	35.83	0.438	0.355	0.448	2.510	2.533	2.440
4 wt%	35.57	35.39	36.12	0.472	0.359	0.402	2.528	2.531	2.428
6 wt%	35.60	35.39	35.36	0.304	0.441	0.462	2.520	2.534	2.530
8 wt%	35.15	35.39	35.44	0.696	0.463	0.446	2.558	2.537	2.538
10 wt%	35.00	35.52	35.12	0.586	0.449	0.355	2.569	2.539	2.549

**Table 3:** XRD parameters for Al:LZO thin films

Material composition Al:LZO (wt%)	Lattice constants (Å)						Internal strain ‘*ε*’			Dislocation density ‘*δ*’ (10^18^ lines per m^2^)		
	200–300 nm		400–500 nm		700–800 nm		200–300 nm	400–500 nm	700–800 nm	200–300 nm	400–500 nm	700–800 nm
	*a* (Å)	*c* (Å)	*a* (Å)	*c* (Å)	*a* (Å)	*c* (Å)						
0 wt%	3.51	5.07	3.52	5.14	3.52	5.07	0.005	0.004	0.005	0.002	0.001	0.003
2 wt%	3.49	5.03	3.48	5.85	3.51	4.97	0.005	0.004	0.005	0.002	0.001	0.003
4 wt%	3.50	5.04	3.51	5.0	3.55	4.93	0.006	0.004	0.007	0.003	0.001	0.005
6 wt%	3.49	5.04	3.50	5.03	3.52	5.07	0.004	0.006	0.007	0.003	0.002	0.006
8 wt%	3.54	5.10	3.52	5.14	3.52	5.06	0.006	0.006	0.008	0.003	0.003	0.005
10 wt%	3.55	5.12	3.24	5.13	3.52	5.08	0.009	0.006	0.005	0.004	0.002	0.00

### 3.2 Surface morphological studies

**Fig. 2:**
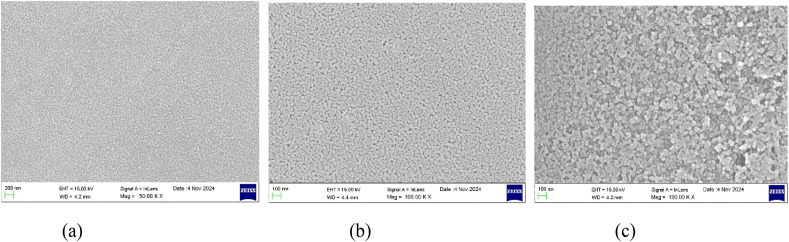
FESEM images of undoped ZnO films of thickness: (a) 200–300 nm, (b) 400–500 nm and (c) 700–800 nm.

**Fig. 3:**
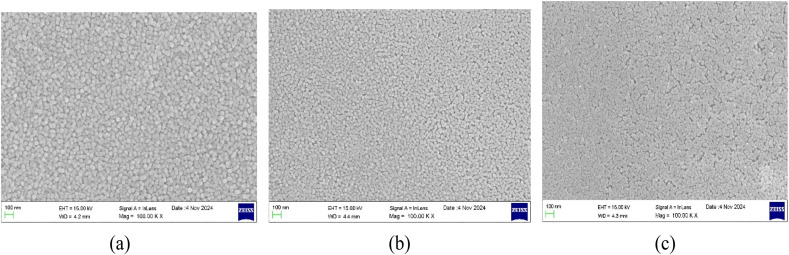
FESEM images of 2 wt% Al:LZO thin films of thickness: (a) 200–300 nm, (b) 400–500 nm and (c) 700–800 nm.

**Fig. 4:**
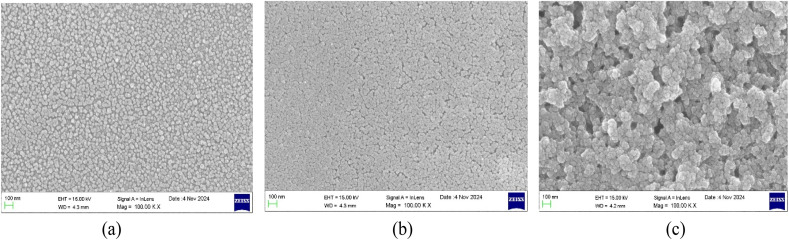
FESEM images of 10 wt% Al:LZO thin films of thickness: (a) 200–300 nm, (b) 400–500 nm and (c) 700–800 nm.

**Fig. 5:**
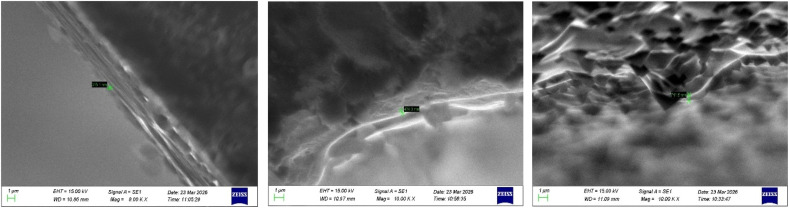
FESEM cross-section images of Al:LZO thin films.

The surface morphologies of the deposited thin films with varying thicknesses were studied using FESEM images. The FESEM images of the undoped, Al 2 wt% LZO and Al 10 wt% LZO thin films of 200–300 nm, 400–500 nm and 700–800 nm thicknesses are shown in Fig. 2–4. The thickness of the deposited films was determined from the cross-sectional scanning electron microscopy (SEM) images seen in Fig. 5. The thickness values were estimated by measuring the distance between the film surface and the substrate interface. Thickness can significantly modify the nonlinear optical response by altering the effective interaction length between the incident light and the material. The images reveal that the film surfaces are free of voids and display a densely packed arrangement with uniform particle sizes. Notably, the surface morphology changes as the film thickness varies. While most films exhibit a dense granular texture composed of well-defined grains, the sample with a thickness of 200–300 nm demonstrates a comparatively smooth surface. Additionally, the grain size on the film surface increases progressively with greater thickness. These observations correlate with the XRD results, which show a decrease in the full width at half maximum (FWHM) as the film thickness increases. As the thickness increases to 400–500 nm, the surface morphology improves notably, with densely packed, uniformly distributed grains and minimal porosity. The grains appear larger and more interconnected, reflecting enhanced crystallinity and structural uniformity. This optimized morphology at 400–500 nm supports superior optical and nonlinear properties, making it the most favourable thickness among the samples. However, a further increase in thickness to 700–800 nm results in grain agglomeration and irregular distribution, accompanied by signs of structural disorder and increased surface roughness. The excessive thickness likely introduces internal stress, leading to defects such as microcracks or voids, thereby reducing the overall film quality. Hence, the 400–500 nm thick film demonstrates the most compact and uniform surface morphology, indicating it as the optimal thickness for achieving high-quality Al–La co-doped ZnO thin films.

### 3.3 Elemental analysis- EDAX

**Fig. 6:**
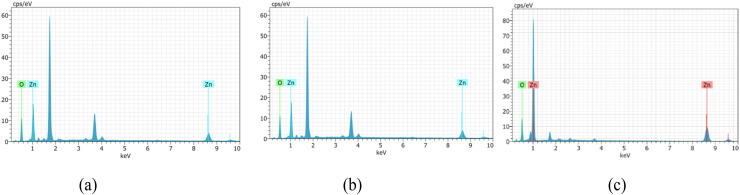
EDAX analysis of undoped ZnO thin films of thickness: (a) 200–300 nm, (b) 400–500 nm and (c) 700–800 nm.

**Fig. 7:**
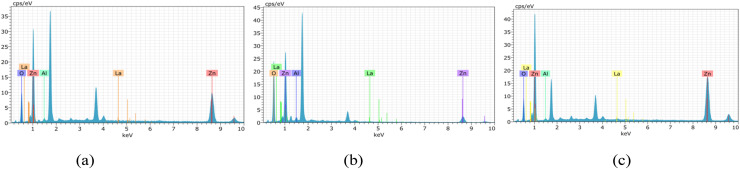
EDAX analysis of 2 wt% Al:LZO of thickness: (a) 200–300 nm, (b) 400–500 nm and (c) 700–800 nm.

**Fig. 8:**
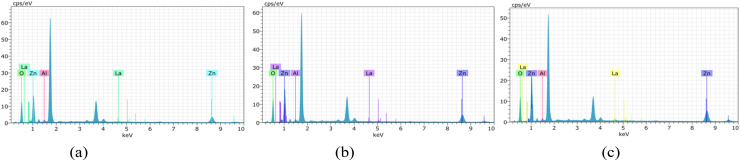
EDAX analysis of 10 wt% Al:LZO of thickness: (a) 200–300 nm, (b) 400–500 nm and (c) 700–800 nm.

The EDAX analysis confirms the successful incorporation of aluminium (Al) and lanthanum (La) into the ZnO thin film matrix. The spectra exhibit prominent peaks corresponding to zinc (Zn) and oxygen (O), verifying the primary composition of ZnO (Fig. 6–8). In addition, the presence of Al and La peaks, even at lower intensities, clearly indicates effective doping without introducing any secondary or impurity phases.

The weight percentage and atomic percentage values obtained from EDAX, shown in Tables 4 and 5, are consistent with the intended doping concentrations, suggesting uniform distribution of the dopant elements across the film surface. The absence of unwanted elements further confirms the chemical purity of the deposited films. Overall, the EDAX results validate the compositional homogeneity and confirm the successful co-doping of ZnO thin films with Al and La.

**Table 4:** Chemical compositions of Al, La: ZnO thin films in wt%

Materials Al:LZO (wt%)	Components (wt%)											
	Zn			La			Al			O		
	200–300 nm	400–500 nm	700–800 nm	200–300 nm	400–500 nm	700–800 nm	200–300 nm	400–500 nm	700–800 nm	200–300 nm	400–500 nm	700–800 nm
0 wt%	60.28	81.38	80.18	0	0	0	0	0	0	39.72	18.62	19.82
2 wt%	76.59	76.24	85.08	0.12	1.23l	1.75	2.97	3.95	1.91	20.32	18.58	11.26
10 wt%	47.70	70.08	69.46	2.43	7.53	6.05	6.86	3.06	3.91	43.01	19.33	20.58

**Table 5:** Chemical compositions of Al, La: ZnO thin films in at%

Materials Al:LZO (wt%)	Components (at%)											
	Zn			La			Al		O			
	200–300 nm	400–500 nm	700–800 nm	200–300 nm	400–500 nm	700–800 nm	200–300 nm	400–500 nm	700–800 nm	200–300 nm	400–500 nm	700–800 nm
0 wt%	27.08	48.59	49.89	0	0	0	0	0	0	72.92	51.41	50.10
2 wt%	45.89	47.50	62.44	0.03	0.23	0.66	4.31	7.03	3.12	45.89	45.24	33.78
10 wt%	19.77	40.80	63.80	0.47	3.30	0.94	6.89	5.75	5.88	72.86	50.15	29.38

### 3.4 AFM – surface topography

**Fig. 9:**
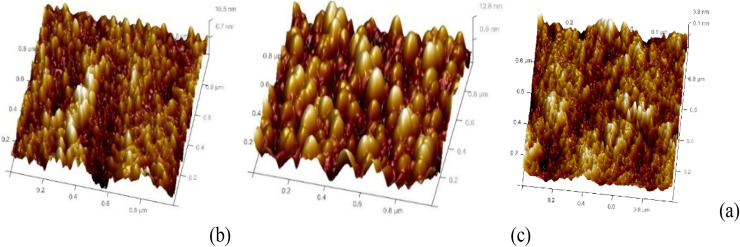
3D AFM images of undoped ZnO thin films of thickness: (a) 200–300 nm, (b) 400–500 nm and (c) 700–800 nm.

**Fig. 10:**
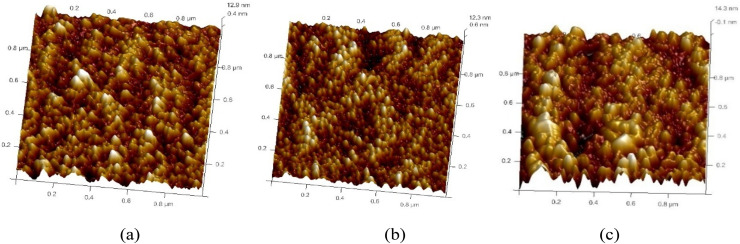
3D AFM images of 2 wt% Al:LZO films of thickness: (a) 200–300 nm, (b) 400–500 nm and (c) 700–800 nm.

**Fig. 11:**
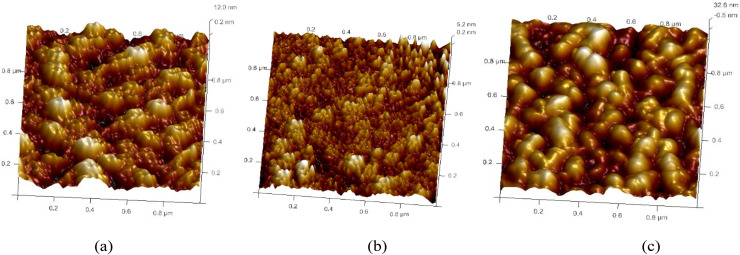
3D AFM images of 10 wt% Al:LZO films of thickness: (a) 200–300 nm, (b) 400–500 nm and (c) 700–800 nm.

Atomic force microscopy (AFM) in tapping mode was employed to investigate the surface topographies of the annealed films. Fig. 9–11 present the AFM images of ZnO films with varying thicknesses, each scanned over an area of 2.0 × 2.0 µm^2^. The images reveal that the surface morphology is predominantly characterized by hexagonally faceted columnar grains. It is evident that the surface roughness increases with film thickness. For instance, the root mean square (RMS) roughness for the film thickness of 200–300 nm was measured to be 2.82 nm, while for a thicker film of 700–800 nm, the RMS roughness progressively rose to 3.91 nm. This increase in roughness is attributed to the growth of larger grains as well as enhanced porosity within the films.^30^ Film growth typically progresses through several distinct stages: nucleation, crystal formation, and grain development. As the film grows, adjacent crystallites compete to establish a preferred orientation (Table 6).^16^

**Table 6:** Average roughness values of Al:LZO thin films

Sample composition wt%	Roughness (*R*_a_)(nm)		
	200–300 nm	400–500 nm	700–800 nm
Undoped ZnO	3.35	2.82	3.58
Al, 2 wt% La:ZnO	3.58	2.61	3.82
Al, 10 wt% La:ZnO	3.66	3.14	3.91

### 3.5 Optical properties

**Fig. 12:**
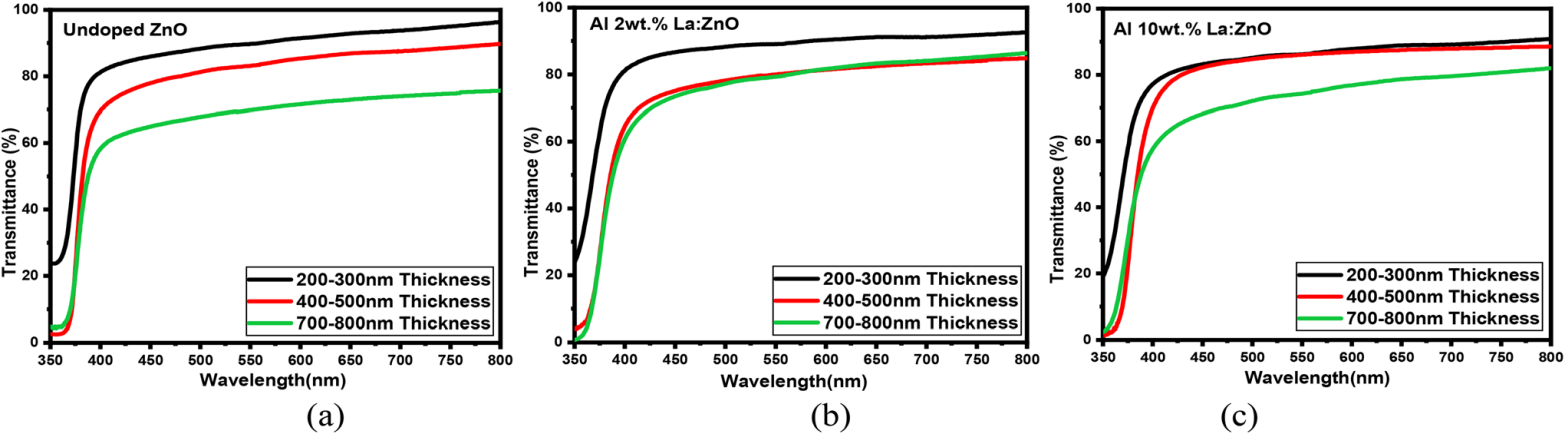
Transmittance (a.u.) *vs.* wavelength (nm) of (a) undoped, (b) Al 2 wt% La:ZnO and (c) Al 10 wt% La:ZnO thin films.

**Fig. 13:**
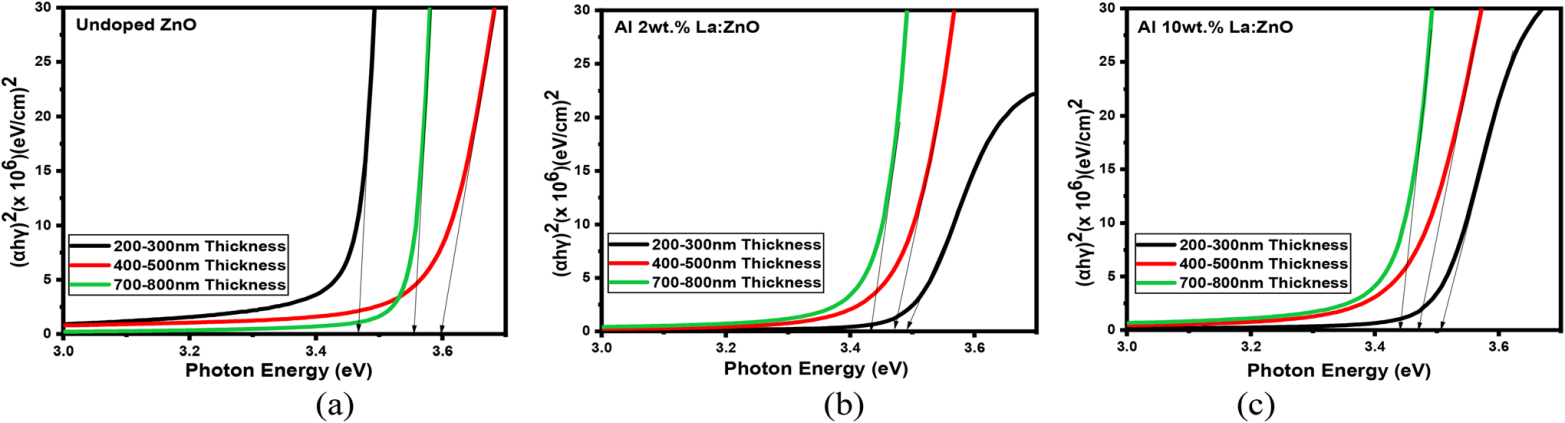
(*αhν*) ^2^*vs. hγ* of (a) undoped, (b) Al 2 wt% La:ZnO and (c) Al 10 wt% La:ZnO thin films.

The transmittance and absorbance spectra as a function of wavelength, shown in Fig. 12 and S2, were obtained through optical spectroscopy. The transmittance spectra of 4, 6, and 8 wt% Al–La co-doped ZnO thin films with varying thicknesses are presented in Fig. S3. The ZnO thin films exhibit high transmittance (>80%) in the visible region and nearly complete absorption in the ultraviolet range. A slight decrease in average transmittance is observed with increasing film thickness. Notably, at higher thicknesses, the films tend to become nearly opaque in the near-infrared region. This behaviour is attributed to an elevated carrier concentration which enhances photon absorption.^17^Fig. 13 depicts the variation of optical band gap as a function of film thickness. The optical band gap plots of 4, 6, and 8 wt% Al–La co-doped ZnO thin films with varying thicknesses are presented in Fig. S4. Variations in crystallite size, lattice strain, and defect density can influence the electronic states near the band edges, leading to a slight shift in the optical band gap. Furthermore, changes in film density and structural uniformity with increasing thickness may also affect the optical absorption behaviour of the films. It is evident that the band gap increases with increasing thickness. This blue shift in the absorption edge is primarily attributed to the Burstein–Moss effect,^18,19^ wherein a higher carrier concentration leads to a shift of the absorption edge toward shorter wavelengths.^20^ The observed widening of the band gap may also be associated with a reduction in the width of the band tail.^21^

**Fig. 14:**
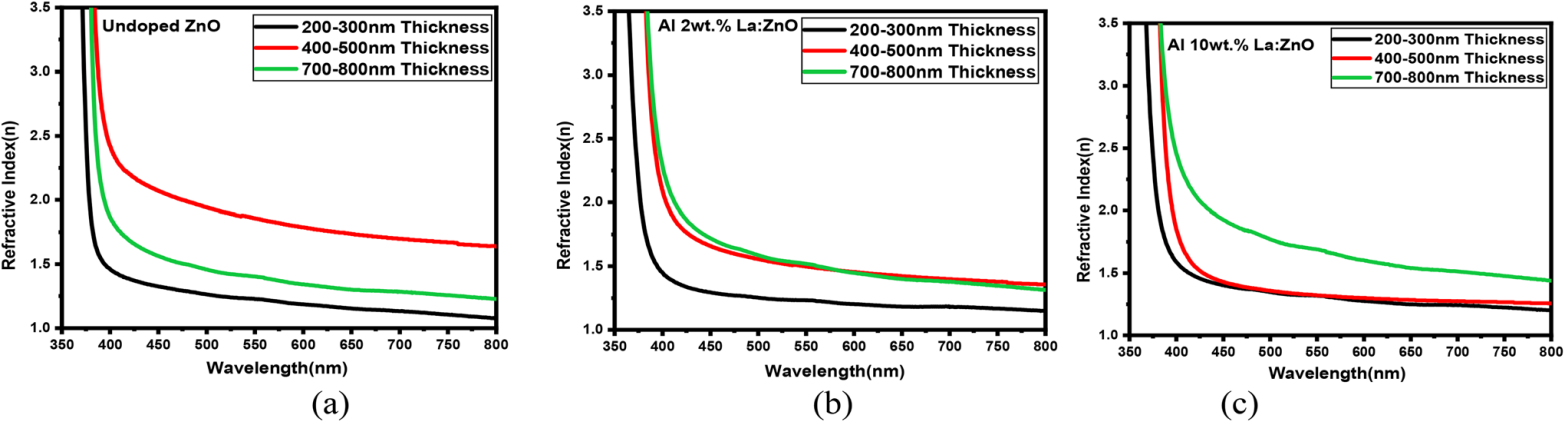
Refractive index (R.I.) *vs.* wavelength (*λ*) of (a) undoped, (b) Al 2 wt% La:ZnO and (c) Al 10 wt% La:ZnO thin films.

The refractive indexes (*n*) of the Al and La co-doped ZnO thin films varied with film thickness, as depicted in Fig. 14, indicating changes in film density and optical uniformity. The refractive indexes (*n*) of 4, 6, and 8 wt% Al–La co-doped ZnO thin films with varying thicknesses are presented in Fig. S5. At 200–300 nm thickness, the refractive index was relatively low, which can be attributed to a less compact microstructure, possible porosity, and surface irregularities resulting from limited growth time. This lower *n*-value suggests weaker optical confinement and reduced interaction with incident light. In the 400–500 nm thick film, the refractive index reached an optimal level, implying improved film densification and crystallinity. The enhanced optical density at this thickness favours efficient light propagation and better structural uniformity. However, a slight decline in the refractive index was observed at 700–800 nm, potentially due to increased surface roughness, grain boundary effects, or internal stress associated with thicker films. This reduction may adversely affect optical performance consistency.^22^ Among the samples, the 400–500 nm film exhibited the most favourable refractive index characteristics for high-quality optical applications.

### 3.6 Photoluminescence properties

**Fig. 15:**
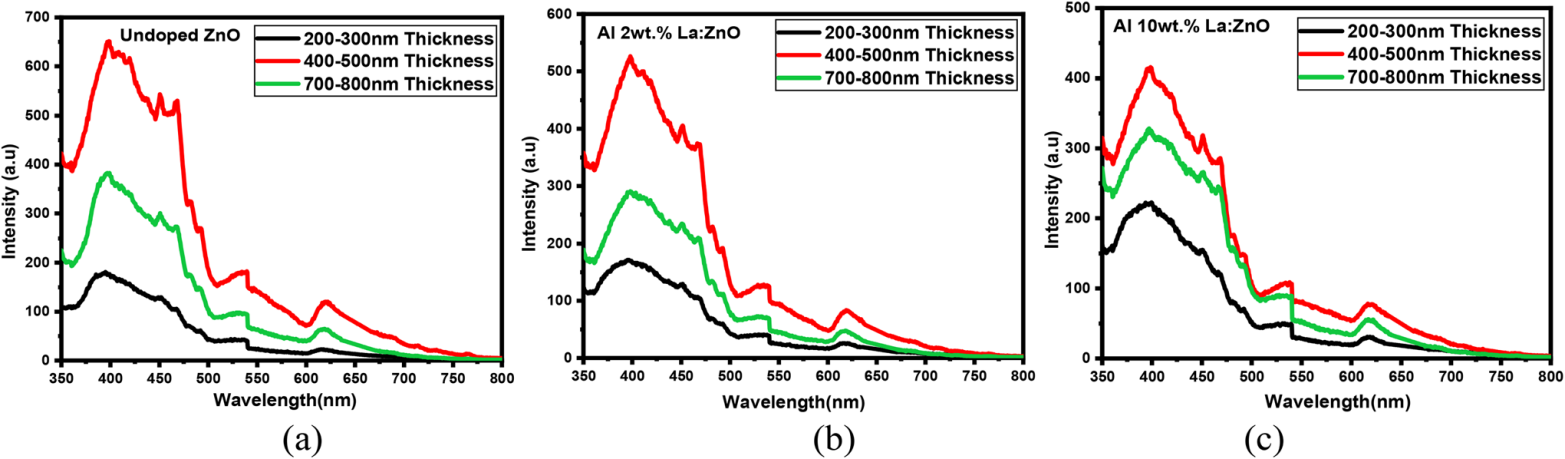
PL intensity *vs.* wavelength of (a) undoped, (b) Al 2 wt% La:ZnO and (c) Al 10 wt% La:ZnO thin films.

To assess the optical behaviour of ZnO films with varying thicknesses, photoluminescence (PL) measurements were conducted, and the results are presented in Fig. 15(a–f). The PL spectra of 4, 6, and 8 wt% Al–La co-doped ZnO thin films with varying thicknesses are presented in Fig. S6. The PL analyses were conducted at room temperature with an excitation wavelength of 325 nm. All samples exhibited a near-band-edge (NBE) emission around 3.27 eV and a broad deep-level emission (DLE) near 2.5 eV. The NBE emission is attributed to excitonic recombination involving donor states, while the DLE is typically linked to intrinsic defects such as oxygen being almost entirely relaxed.^23^

In the 200–300 nm film, the PL spectrum was characterized by a relatively strong deep-level emission (DLE) alongside a weaker near-band-edge (NBE) emission, indicating the presence of a high density of intrinsic defects, such as oxygen vacancies and zinc interstitials, often resulting from limited grain growth and incomplete crystallization during shorter deposition times. As the thickness increased to 400–500 nm, the DLE intensity significantly decreased while the NBE emission became more pronounced and sharper, suggesting a reduction in defect-related non-radiative recombination centers and improved crystalline order.^24^ This enhancement is attributed to the optimized film morphology and better incorporation of dopants at this intermediate thickness, which minimizes defect formation. In contrast, the 700–800 nm thin film showed a slight resurgence in DLE intensity and broadening of the NBE peak, which may be attributed to defect accumulation, increased surface roughness, or internal stress associated with prolonged growth. These findings confirm that the 400–500 nm thick Al and La co-doped ZnO film exhibits the most favourable photoluminescence characteristics, with a high NBE-to-DLE intensity ratio, making it particularly suitable for optoelectronic and light-emitting applications.^25^

### 3.7 Third-order nonlinear optical (TONLO) properties

Nonlinear optical (NLO) effects arise from the interaction between a high-intensity laser pulse and the electronic structure of a material, leading to the induction of electric polarization.^26^ This induced polarization is fundamental to the production of higher-order harmonics, as it alters the primary frequency of the incident light. In this work, the 3rd-order NLO characteristics of Al and LZO films were investigated by single-beam Z-scan technique. This approach is widely recognized as one of the most straightforward and effective methods for examining third-order nonlinear optical responses. It provides the capability not only to detect nonlinear effects but also to distinguish and quantify the individual contributions of NLA and NLR. More generally, materials with NLO characteristics can be studied through a variety of methods, such as three-wave mixing,^27^ degenerate four-wave mixing,^28^ the Kerr effect,^29^ ellipsometry rotation,^30^ interferometry,^31^ beam self-deflection,^32^ third-harmonic generation,^33^ and two-photon fluorescence,^34^ as well as photothermal,^35^ photoacoustic^36^ and beam distortion measurement techniques.^37^

The Z-scan technique, initially introduced by Sheik-Bahae,^38^ is widely used to measure both the magnitude and sign of the nonlinear refractive index (NLR) as well as the nonlinear absorption coefficient of materials, all within a single-beam setup. This method involves moving the sample along the propagation axis of a focused Gaussian laser beam while monitoring the light intensity. Interaction of the beam of the laser with the nonlinear medium causes variations in both the amplitude and phase of the beam, reflecting the material's nonlinear optical behaviour. In the closed-aperture (CA) Z-scan configuration, an aperture placed in the far field relative to the focal point is employed to detect the transmitted light. This setup minimizes the influence of nonlinear absorption, thereby enabling selective measurement of nonlinear refraction by being primarily sensitive to phase changes in the beam. Conversely, the open-aperture (OA) Z-scan method removes the aperture, allowing the transmitted signal to capture the combined effects of NLA and NLR. The OA configuration is particularly useful for extracting the nonlinear absorption coefficient and assessing the overall nonlinear optical response of the material. Ultimately, Z-scan measurements enable determination of the effective third-order nonlinear susceptibility, which encompasses both the real (refractive) and imaginary (absorptive) components.

The parameters employed in the current study are summarized in Tables 7 and 8. The laser beam waist radius (*ω*_0_) at the focal plane was calculated to be 3.8740 × 10^−5^ m, resulting in a peak intensity (I_0_) at the focus of 8.48 × 10^7^ W m^−2^. The waist radius was determined using the expression *ω*_0_ = 4*λf*/(π*d*), where *d* represents the beam diameter, *f* is the focal length of the lens, and *λ* is the laser wavelength. The Rayleigh range (*Z*_0_) associated with the focused laser beam was found to be 8.86 mm, computed by the formula 
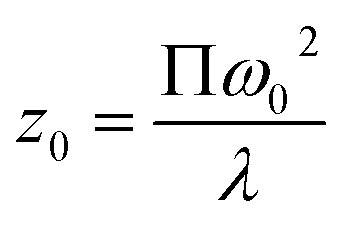
. The Rayleigh range corresponds to the distance from the beam waist to the point along the propagation axis where the cross-sectional area of the beam doubles.

**Table 7:** Third-order nonlinear optical parameters of Al:LZO thin films

Materials Al:LZO (wt%)	*B* × 10^−4^ (cm W^−1^)			*n*_2_ × 10^−12^ (cm^2^ W^−1^)		
	200–300 nm	400–500 nm	700–800 nm	200–300 nm	400–500 nm	700–800 nm
Undoped ZnO	0.22	0.82	0.82	0.71	0.84	0.84
Al 2wt% LZO	0.39	0.79	0.89	1.34	0.21	1.28
Al 4wt% LZO	2.42	2.62	2.57	1.32	2.34	2.28
Al 6wt% LZO	3.89	4.79	4.89	0.98	2.96	2.84
Al 8wt% LZO	2.37	2.24	2.51	1.24	1.28	1.36
Al 10wt% LZO	1.26	1.34	1.26	1.17	1.24	1.17

**Table 8:** Third-order nonlinear susceptibility parameters of Al:LZO thin films

Sample composition wt%	Real part *χ*^(3)^_R_ ×10^−5^ (esu)			Imag. part (*χ*^(3)^_img_) ×10^−5^ esu			Third order *χ*^3^ ×10^−5^ (esu)		
	200–300 nm	400–500 nm	700–800 nm	200–300 nm	400–500 nm	700–800 nm	200–300 nm	400–500 nm	700–800 nm
Undoped ZnO	1.01	1.17	1.17	1.13	1.39	1.59	1.17	1.57	1.97
Al 2wt% LZO	1.32	1.22	2.32	1.18	1.38	1.98	1.45	1.65	3.05
Al 4wt% LZO	2.47	4.68	4.47	2.05	3.15	2.15	2.96	5.46	4.96
Al 6wt% LZO	1.84	4.81	4.84	1.81	3.76	4.81	1.82	6.46	6.82
Al 8wt% LZO	1.11	3.07	3.11	1.84	3.14	3.84	2.38	3.78	4.38
Al 10wt% LZO	1.02	1.16	1.02	1.50	1.31	1.50	1.61	1.51	1.81

A third-order nonlinear optical response can give rise to phenomena such as third-harmonic generation (THG) and two-photon absorption (TPA). The intensity-dependent refractive index is described by the relation^39^

7 *n* = *n*_o_ + *n*_2_*I*

8
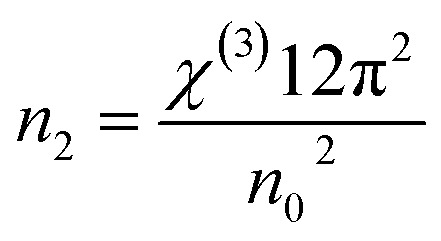
where *n*_2_ is the coefficient of nonlinear refractive index and *I* is the laser light intensity. The thin film sample's linear refractive index is represented by *n*_o_.

#### 3.7.1 Nonlinear absorption

The nonlinear absorption coefficient of a material is determined through the open-aperture Z-scan technique. This method can reveal both saturable absorption (SA) and reverse saturable absorption (RSA) phenomena. RSA occurs when two-photon absorption (TPA) predominates. The presence of RSA is indicated by a minimum in the transmission at the focal point. Conversely, saturable absorption is characterized by a peak in normalized transmittance near the focus, where the transmission increases as the incident light intensity rises.

The value of the nonlinear absorption coefficient (B_eff_) is calculated from open aperture Z-scan data. It is given by eqn (9).^40^9
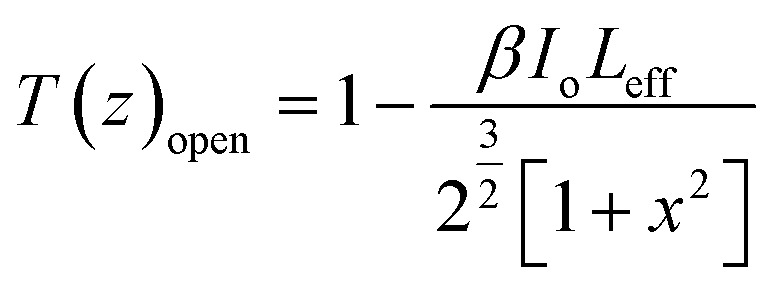


**Fig. 16:**
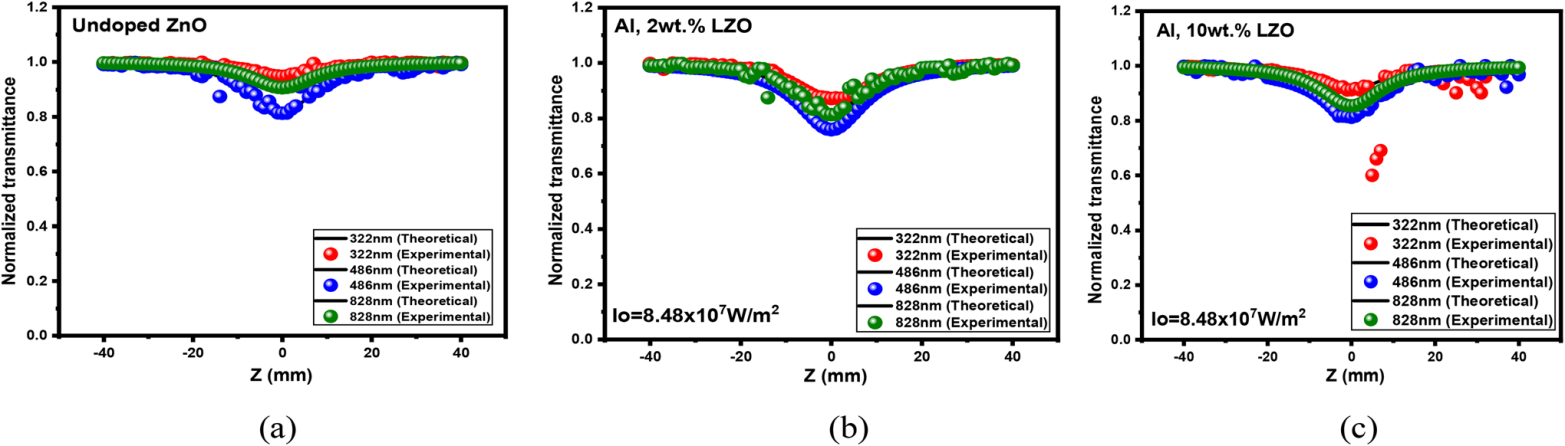
OA traces of (a) undoped, (b) Al 2 wt% La:ZnO and (c) Al 10 wt% La:ZnO thin films.

The NLO behaviour of Al, La co-doped ZnO thin films was investigated using both open and closed aperture Z-scan techniques, which revealed a strong dependence on film thickness. In the open aperture configuration, seen in Fig. 16, all films exhibited reverse saturable absorption (RSA), confirming a positive nonlinear absorption coefficient (*β*). The OA curves of 4, 6, and 8 wt% Al–La co-doped ZnO thin films with varying thicknesses are presented in Fig. S7. The 200–300 nm film showed a weaker RSA response due to limited optical path length and higher density of surface defects which restricted efficient photon–defect interaction. In contrast, the 400–500 nm film demonstrated a pronounced RSA effect, indicating enhanced third-order nonlinear absorption. This improvement is attributed to increased film thickness providing a longer interaction length, improved crystallinity, and reduced defect states, which together facilitate multi-photon absorption mechanisms. The 700–800 nm thick film, although showing RSA behaviour, exhibited a reduction in nonlinear absorption strength, likely due to increased scattering, dopant clustering, and stress-induced imperfections at higher thickness.

#### 3.7.2 Nonlinear refractive index (NLR)

Closed-aperture (CA) Z-scan measurements are primarily based on self-phase modulation and self-refraction effects.^41^ This technique produces a closed aperture at the far field, enabling the detector to measure the sample's transmittance as a function of its position along the *Z*-axis (in millimetres). The CA Z-scan response differs depending on whether the material exhibits positive or negative nonlinearity, with each showing opposite behaviours. When nonlinear absorption is negligible, this behaviour is referred to as purely refractive nonlinearity. Multiphoton absorption (MPA) tends to increase the valley depth and diminish the peak height in the transmittance curve.^42^ Conversely, saturable absorption causes the opposite effect, reducing the valley and enhancing the peak.

The nonlinear phase shift (Δ*ϕ*_o_) can be calculated by fitting the CA data using the following equation.^43^10
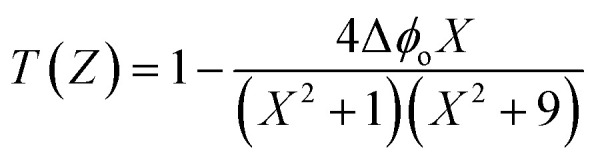
Here, 
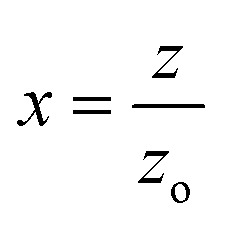
 where *Z*_o_ is the Rayleigh length and *Z* is the sample's longitudinal displacement from the focal point.

The nonlinear refractive index can be calculated from the following equation.^44^11
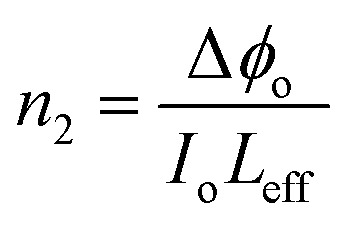


Third-order nonlinear susceptibility is a complex quantity is given by eqn (12),^45^

12 |*χ*^(3)^| = |*χ*^(3)^_R_ + *χ*^(3)^_Im_|

where the real and imaginary parts are represented by *χ*^(3)^_R_ and *χ*^(3)^_Im_, respectively.^46^13
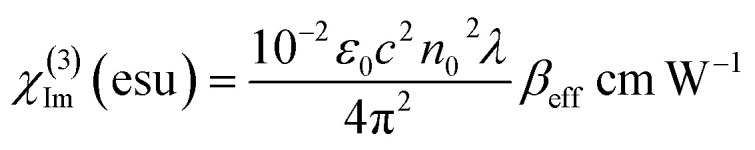
14
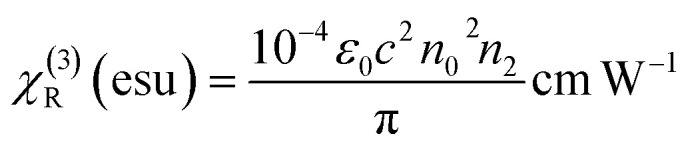


The absolute value of the third-order nonlinear susceptibility is given by eqn (15).^47^15
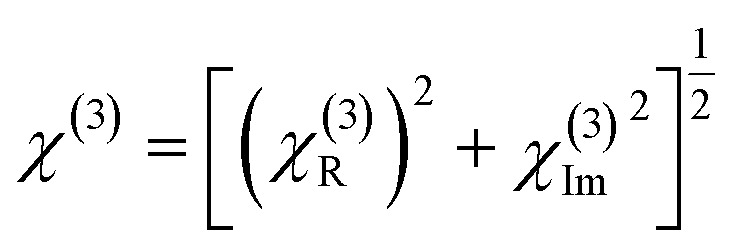


**Fig. 17:**
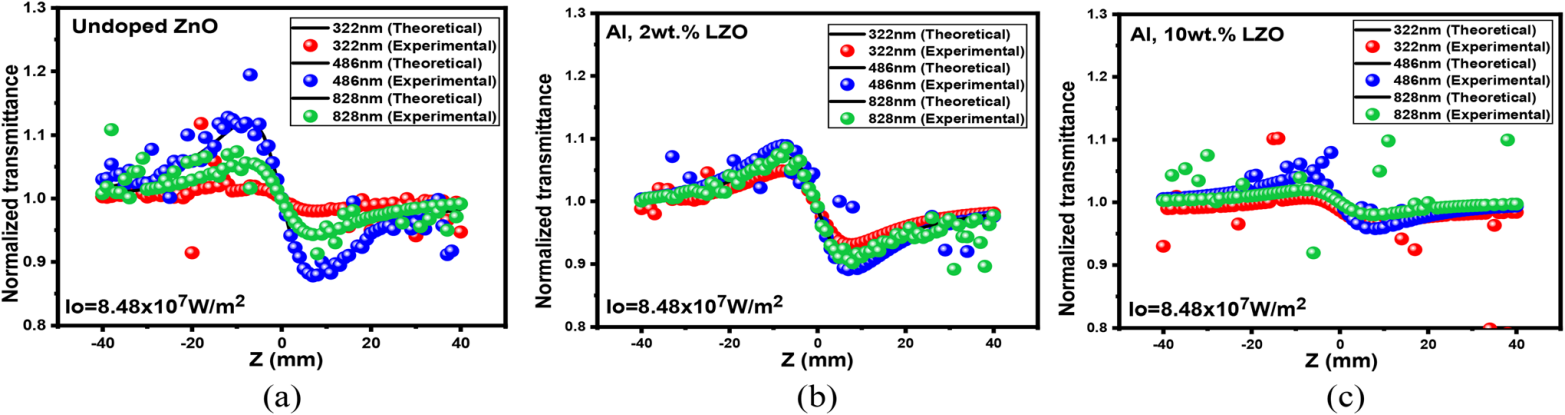
CA traces of (a) undoped, (b) Al 2 wt% La:ZnO and (c) Al 10 wt% La:ZnO thin films.

Fig. 17 presents the closed-aperture (CA) Z-scan results for the Al:La co-doped ZnO (LZO) films. The CA curves of 4, 6, and 8 wt% Al–La co-doped ZnO thin films with varying thicknesses are presented in Fig. S8. The nonlinear refractive index (NLR.I) *n*_2_ calculated from the closed aperture data indicates that the deposited films show a self-defocusing nature alongside a positive refractive index. This effect is evident at doping levels of 0, 2, 4, 6, 8 and 10 wt%, where the characteristic transmittance curve shows a valley followed by a peak. The variations observed in the nonlinear refractive index values throughout this study are primarily attributed to thermal effects caused by the continuous laser irradiation of the films.

Among the three thickness ranges, the Z-scan CA results of the 400–500 nm thickness film showed the strongest peak–valley separation in the closed aperture scan, reflecting the highest magnitude of nonlinear refractive index. This can be correlated with optimized dopant incorporation and improved structural uniformity, which enhance nonlinear refraction.^48^ Therefore, the 400–500 nm thick Al and La co-doped ZnO thin film exhibits superior performance in both nonlinear absorption and nonlinear refraction, making it the most suitable for optical limiting devices.

The nonlinear optical response of ZnO thin films is strongly influenced by their microstructural properties. Changes in grain size, crystallinity, and surface morphology can significantly modify defect states, thereby influencing the interaction between the incident electromagnetic field and the electronic structure of the material, which ultimately affects the third-order nonlinear optical coefficients. The 3rd-order susceptibility, *χ*^(3)^, of Al:LZO films is strongly influenced by the material's structural symmetry. As shown in Table 8, *χ*^(3)^ increases with La doping up to 6 wt% in the 400–500 nm thickness. This enhancement in nonlinear behaviour is attributed to improved crystallinity resulting from increased doping concentration.^49^ La incorporation modifies the refractive index and the film's microstructure, thereby enhancing light trapping within the thin film. This effect boosts the local photon density, promoting multiphoton absorption processes and consequently elevating the nonlinear response.^50^ The measured values of the nonlinear absorption coefficient *β* and nonlinear R.I. are reported in Table 7. The calculated (*χ*3) values in Table 8 confirm that the NLO properties of co-doped films with a thickness range of 400–500 nm are suitable for their application in optoelectronic devices.

### 3.8 Optical limiting properties

**Fig. 18:**
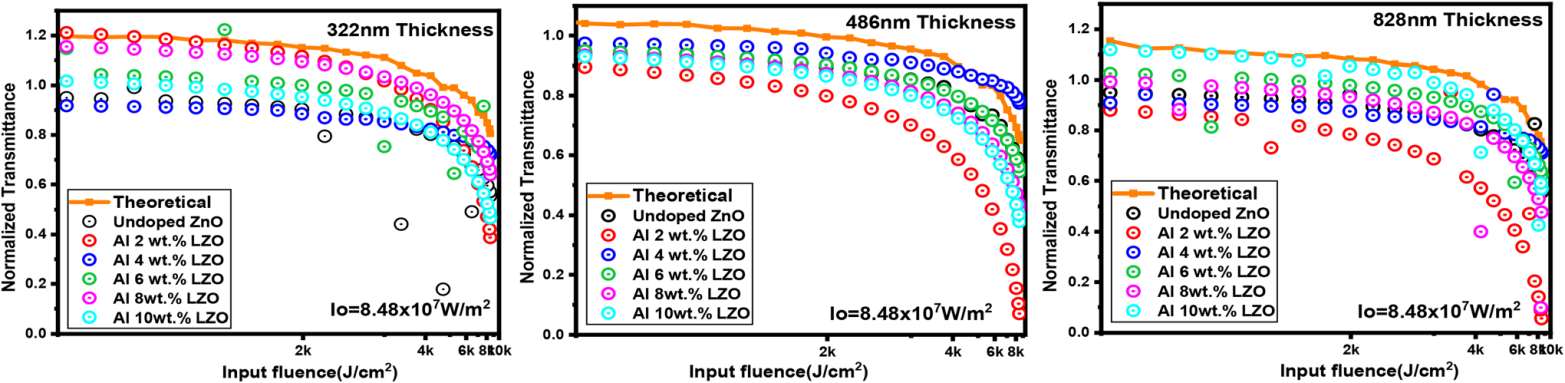
Optical limiting performance of Al:LZO thin films.

The optical limiting measurements were carried out using a Z-scan technique with a continuous-wave (CW) laser operating at a wavelength of 532 nm and an input power of 200 mW. The laser beam had a diameter of 0.7 cm and was focused using a lens with a focal length of 28 cm. During the Z-scan measurements, the sample was translated along the optical axis from −40 mm to +40 mm relative to the focal point. All measurements were performed under room temperature conditions. The optical limiting threshold was determined from the plot of transmitted output intensity as a function of the incident laser intensity. The point at which this deviation from linear transmission occurs is defined as the optical limiting threshold. This threshold represents the minimum incident intensity at which the material starts to limit the transmitted output, thereby protecting sensitive optical sensors and photonic devices from high-intensity laser damage. A lower optical limiting threshold indicates a better optical limiting performance of the material. The increase in optical limiting (OL) performance observed with Al and La co-doping is attributed to the increased nonlinear absorption induced by the dopant ions, as illustrated in Fig. 18. The incorporation of lanthanum into the ZnO matrix likely creates defect states or localized energy levels within the bandgap which facilitate excited-state absorption processes such as reverse saturable absorption (RSA).^51^ This effect is essential for optical limiting because materials exhibiting RSA absorb more light at higher intensities, thereby limiting the transmitted output and protecting sensitive optical components.

The lowest OL threshold of 1.2 kJ cm^−2^, recorded in the 6 wt% La 400–500 nm thickness film indicates an optimal doping level. Beyond this concentration, the optical limiting efficiency may decline, possibly due to dopant-related scattering or phase separation effects. These findings emphasize the importance of precise control over La doping to optimize the nonlinear optical properties of ZnO thin films for applications in laser protection and optical sensor safeguarding. Optical limiting behaviour is directly related to nonlinear absorption processes that contribute to the third-order optical nonlinearity. As the film thickness increases, the effective optical path length within the material also increases, leading to stronger interaction between the incident laser beam and the thin film. This enhanced interaction promotes nonlinear absorption processes, which strengthen the third-order nonlinear optical response and result in a reduction of the optical limiting threshold. However, if the thickness becomes excessively large, optical scattering, increased defect density, and light attenuation may reduce the efficiency of nonlinear interaction. Therefore, an optimal thickness exists where the balance between nonlinear interaction and optical losses produces both a strong third-order nonlinear response and an efficient optical limiting performance. The 200–300 nm thick film displayed weak optical limiting action due to its shorter optical path length and higher density of surface defects, which limited the extent of nonlinear absorption processes necessary for effective optical limiting. In contrast, the 400–500 nm film exhibited a strong optical limiting performance characterized by a more pronounced reduction in transmittance at higher input intensities. This enhanced limiting response is attributed to the optimized microstructural properties at this thickness, including improved crystallinity, uniform dopant incorporation, and efficient reverse saturable absorption (RSA) processes. The moderate thickness ensures sufficient interaction between incident photons and the nonlinear medium, facilitating mechanisms such as excited-state absorption and multi-photon absorption, which are critical for optical limiting. However, the 700–800 nm thick film showed a reduction in limiting efficiency, likely due to increased light scattering, defect accumulation, and saturation effects that hinder effective absorption modulation at high intensities. Overall, the 486 nm thickness Al and La co-doped ZnO thin film demonstrated the most effective optical limiting behaviour, making it highly suitable for applications in laser protection and photonic limiting devices.

## 4. Conclusion

In conclusion, the systematic doping of ZnO thin films of 400–500 nm thickness with aluminium (4 wt%) and lanthanum (6 wt%) led to significant enhancement in their structural, optical, and morphological properties, establishing their suitability for optoelectronic and photonic applications. Thickness variation was found to play a crucial role in tuning these properties. Among the studied thicknesses of 200–300 nm, 400–500 nm, and 700–800 nm, the 486 nm thin film exhibited optimal performance across multiple parameters. It showed improved crystallinity, reduced intrinsic defect states, and a balanced optical bandgap, as evidenced by enhanced near-band-edge emission and suppressed deep-level emissions in photoluminescence spectra. The extinction coefficient and refractive index were also found to be most favourable at this intermediate thickness, indicating an optimal microstructure and better optical confinement. Moreover, nonlinear optical studies confirmed strong reverse saturable absorption and pronounced self-defocusing behaviour in the 486 nm film, along with the highest third-order nonlinear susceptibility and most efficient optical limiting response. In contrast, the 200–300 nm and 700–800 nm films displayed relatively weaker characteristics due to either limited growth or increased scattering and defect accumulation. Overall, this study demonstrates that both dopant optimization and precise thickness control are essential in engineering Al and La co-doped ZnO thin films, making them highly promising for future applications in optoelectronic devices and optical limiters.

## Conflicts of interest

The authors declare that there is no conflict of interest in the current work.

## Supplementary Material

RA-016-D6RA00168H-s001

## Data Availability

The data supporting this article have been included as part of the Supplementary Information (SI). Supplementary information: additional figures (Fig. S1–S8) presenting X-ray diffraction patterns, absorbance and transmittance spectra, optical band gap analysis, refractive index variation, photoluminescence spectra, and open- and closed-aperture Z-scan traces of Al:LZO thin films. See DOI: https://doi.org/10.1039/d6ra00168h.
