# Supplementary material for: Thickness-driven modulation of linear and nonlinear optical properties in La–Al co-doped ZnO thin films for optical limiting applications
Source: RSC Adv. 2026 May 8;16(27):24280–95. doi: 10.1039/d6ra00168h (PMC13154006; doi:10.1039/d6ra00168h)
Supplement: RA-016-D6RA00168H-s001 [file RA-016-D6RA00168H-s001.pdf]

Supplementary Information (SI) for RSC Advances.
This journal is © The Royal Society of Chemistry 2026

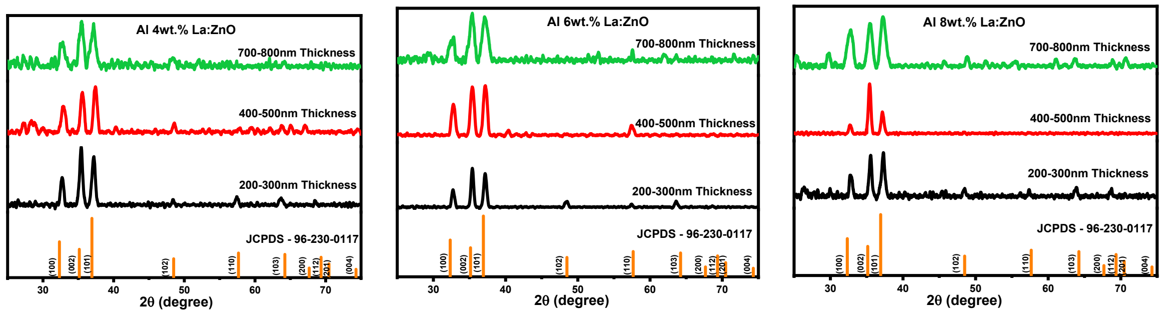

(a) (b) (c)

Fig.S1: X-ray diffraction patterns of 4wt.%, 6wt.% and 8wt.% of Al: LZO film

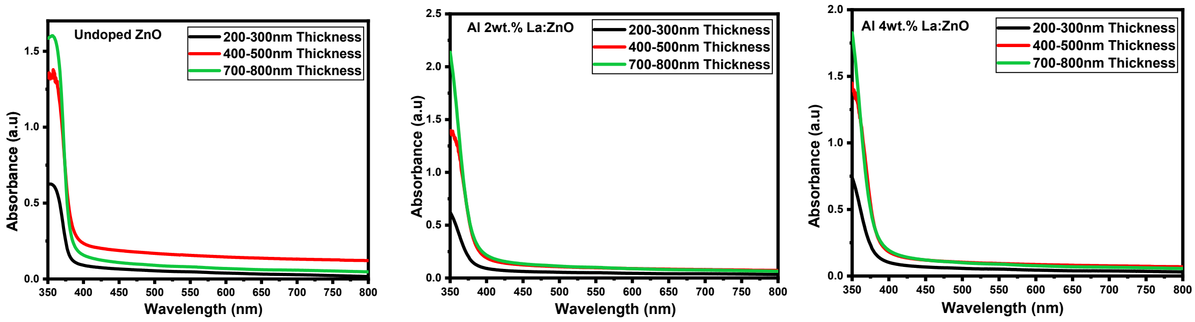

(a) (b) (c)

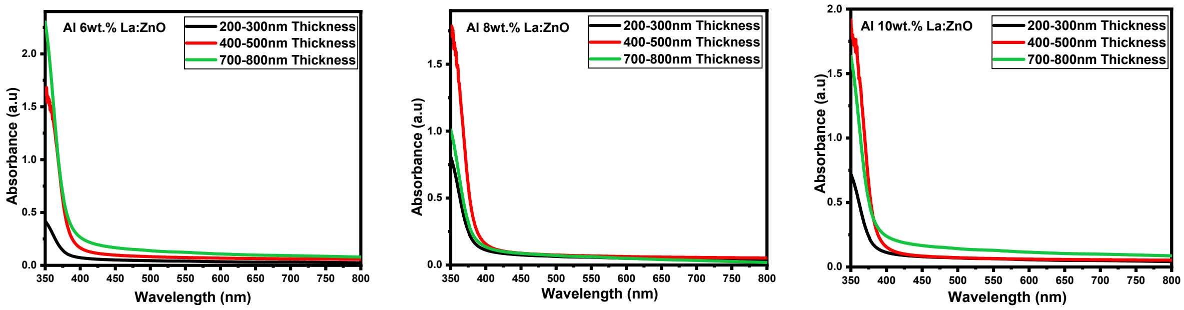

(d) (e) (f)

Fig.S2: Absorbances (a.u) v/s Wavelength (nm) of Al: LZO films of 0-10 wt.% (a-f).

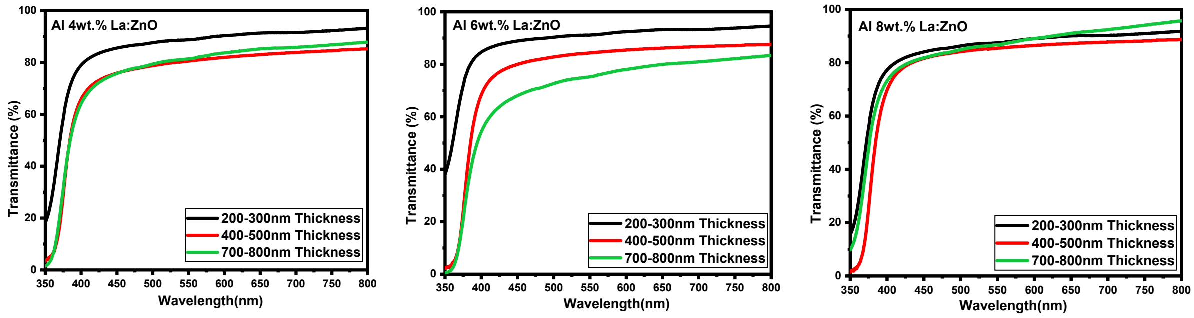

(a) (b) (c)

Fig.S3: Transmittance (a.u) v/s Wavelength (nm) of 4wt.%, 6wt.% and 8wt.% of Al: LZO films

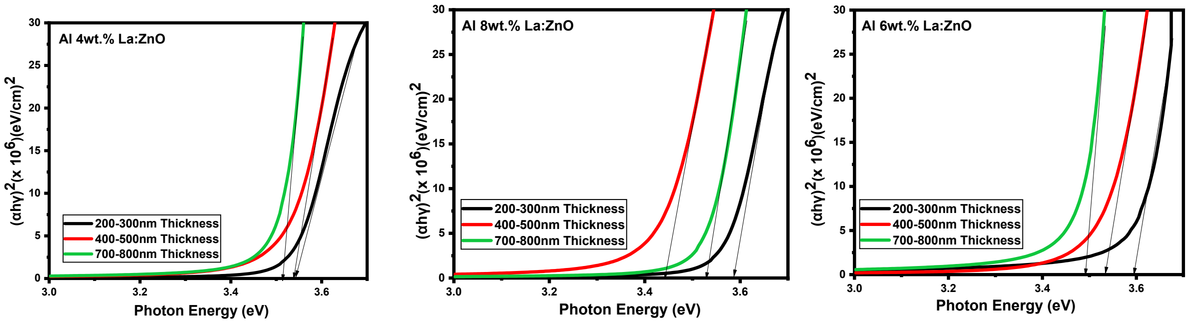

(a) (b) (c)

Fig.S4: (αhν) ² Vs $h\gamma$ of 4wt.%, 6wt.% and 8wt.% of Al: LZO films

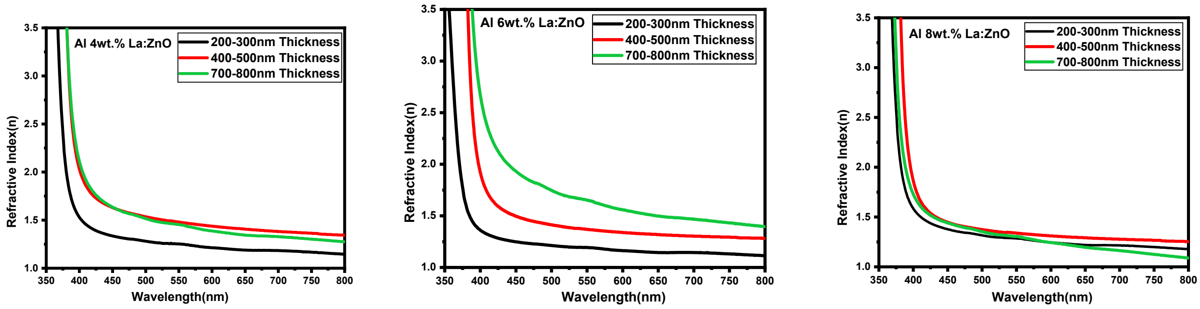

(a) (b) (c)

Fig.S5: Refractive index (R.I) Vs Wavelength (λ) of4wt.%, 6wt.% and 8wt.% of Al: LZO films

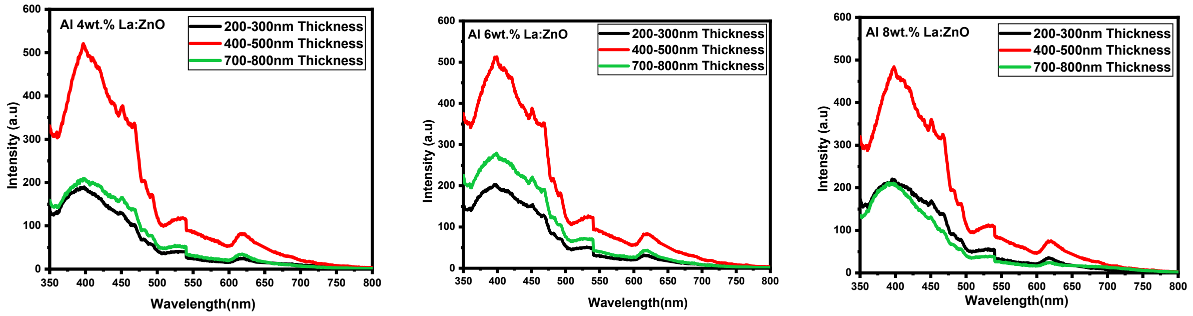

(a) (b) (c)

Fig.S6: PL intensity Vs Wavelength of 4wt.%, 6wt.% and 8wt.% of Al: LZO films

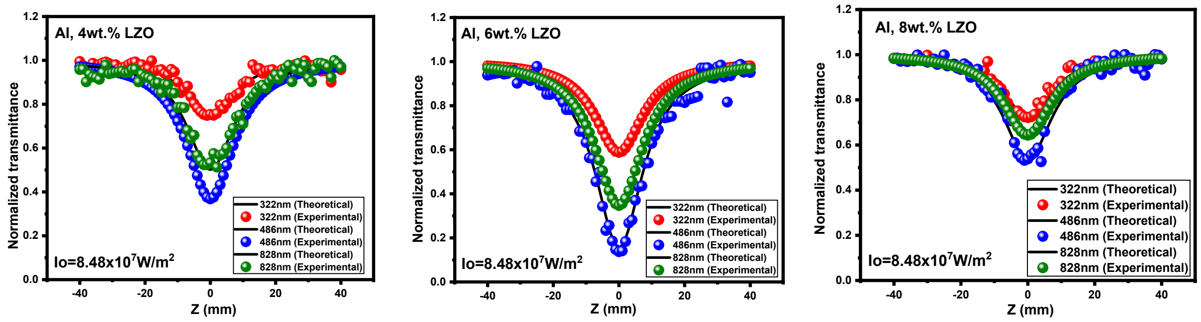

(a) (b) (c)

Fig.S7: OA traces of 4wt.%, 6wt.% and 8wt.% of Al: LZO films

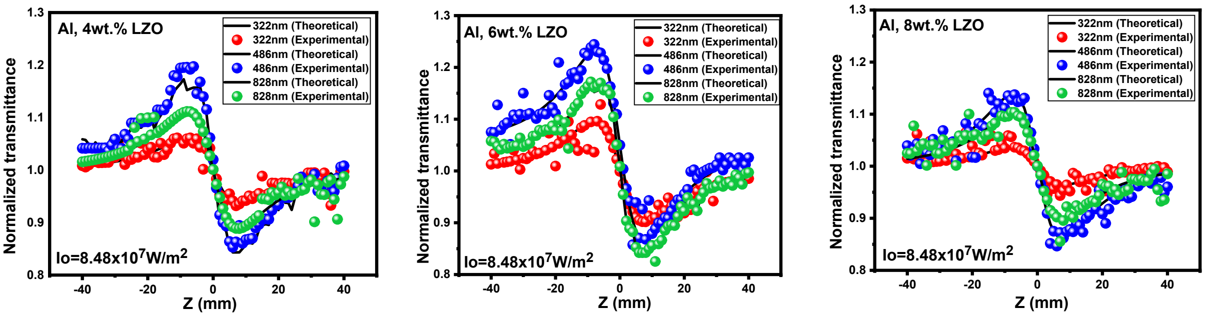

(a) (b) (c)

Fig.S8: CA traces of 4wt.%, 6wt.% and 8wt.% of Al: LZO films
